# Supplementary material for: End Sequence Analysis Toolkit (ESAT) expands the extractable information from single-cell RNA-seq data
Source: Genome Res. 2016 Oct;26(10):1397–410. doi: 10.1101/gr.207902.116 (PMC5052061; doi:10.1101/gr.207902.116)
Supplement: Supplemental Material [file supp_gr.207902.116_Supp_Materials.tar.gz › ESAT-esat_v0.1/doc/html/classumms_1_1core_1_1writers_1_1_paired_end_writer.html]

ESAT: umms.core.writers.PairedEndWriter Class Reference


ESAT


- Main Page
- Related Pages
- Packages
- Classes
- Files

- Class List
- Class Index
- Class Hierarchy
- Class Members

All Classes Namespaces Files Functions Variables Enumerator Pages

- umms
- core
- writers
- PairedEndWriter

Public Member Functions |
Static Public Member Functions |
Static Public Attributes |
List of all members

umms.core.writers.PairedEndWriter Class Reference

Collaboration diagram for umms.core.writers.PairedEndWriter:

[legend]

|  |  |
| --- | --- |
| Public Member Functions | |
|  | PairedEndWriter (File bamFile) |
|  | |
|  | PairedEndWriter (File bamFile, String output) |
|  | |
| void | setMaxAllowableInsert (int x) |
|  | |
| void | convertInputToPairedEnd () |
|  | |
| void | convertInputToPairedEnd (TranscriptionRead txnRead) |
|  | |
| String | getOutputFile () |
|  | |
| void | addAlignment (final Alignment alignment) |
|  | |
| void | addRecord (final SAMRecord record) |
|  | |
| void | close () |
|  | |

|  |  |
| --- | --- |
| Static Public Member Functions | |
| static String | getDefaultFile (String file) |
|  | |

|  |  |
| --- | --- |
| Static Public Attributes | |
| static String | PAIRED\_END\_EXTENSION = ".PairedEnd.bam" |
|  | |
| static final String | mateSequenceFlag ="ms" |
|  | |
| static final String | mateCigarFlag ="mc" |
|  | |
| static final String | readStartFlag ="rs" |
|  | |
| static final String | readCigarFlag ="rc" |
|  | |
| static final String | mateLineFlag ="mateLine" |
|  | |

## Constructor & Destructor Documentation

|  |  |  |  |  |  |
| --- | --- | --- | --- | --- | --- |
| umms.core.writers.PairedEndWriter.PairedEndWriter | ( | File | *bamFile* | ) |  |

Parameters
:   |  |  |
    | --- | --- |
    | bamFile | Input SAM or BAM file to extract header and/or reads from. |

Here is the call graph for this function:

|  |  |  |  |
| --- | --- | --- | --- |
| umms.core.writers.PairedEndWriter.PairedEndWriter | ( | File | *bamFile*, |
|  |  | String | *output* |
|  | ) |  |  |

Parameters
:   |  |  |
    | --- | --- |
    | bamFile | Input SAM or BAM file to extract header and/or reads from. |
    | output | Output path |

## Member Function Documentation

|  |  |  |  |  |  |
| --- | --- | --- | --- | --- | --- |
| void umms.core.writers.PairedEndWriter.addAlignment | ( | final Alignment | *alignment* | ) |  |

Write a single Alignment

Parameters
:   |  |  |
    | --- | --- |
    | alignment |  |

Here is the call graph for this function:

|  |  |  |  |  |  |
| --- | --- | --- | --- | --- | --- |
| void umms.core.writers.PairedEndWriter.addRecord | ( | final SAMRecord | *record* | ) |  |

Here is the caller graph for this function:

|  |  |  |  |  |
| --- | --- | --- | --- | --- |
| void umms.core.writers.PairedEndWriter.close | ( |  | ) |  |

Here is the caller graph for this function:

|  |  |  |  |  |
| --- | --- | --- | --- | --- |
| void umms.core.writers.PairedEndWriter.convertInputToPairedEnd | ( |  | ) |  |

Convert the bamFile provided in the constructor to paired end format. If no transcription read is supplied, set to unstranded

|  |  |  |  |  |  |
| --- | --- | --- | --- | --- | --- |
| void umms.core.writers.PairedEndWriter.convertInputToPairedEnd | ( | TranscriptionRead | *txnRead* | ) |  |

Convert the bamFile provided in the constructor to paired end format. Convert the bamFile provided in the constructor to paired end format.

Here is the call graph for this function:

|  |  |  |  |  |  |
| --- | --- | --- | --- | --- | --- |
| static String umms.core.writers.PairedEndWriter.getDefaultFile | ( | String | *file* | ) |  |

static

Here is the caller graph for this function:

|  |  |  |  |  |
| --- | --- | --- | --- | --- |
| String umms.core.writers.PairedEndWriter.getOutputFile | ( |  | ) |  |

|  |  |  |  |  |  |
| --- | --- | --- | --- | --- | --- |
| void umms.core.writers.PairedEndWriter.setMaxAllowableInsert | ( | int | *x* | ) |  |

## Member Data Documentation

final String umms.core.writers.PairedEndWriter.mateCigarFlag ="mc"

static

final String umms.core.writers.PairedEndWriter.mateLineFlag ="mateLine"

static

final String umms.core.writers.PairedEndWriter.mateSequenceFlag ="ms"

static

Writes a file to represent a BAM file where the paired end reads are concatenated We will represent the basic alignment start and end for the whole fragment The attributes will have information for the component parts

String umms.core.writers.PairedEndWriter.PAIRED\_END\_EXTENSION = ".PairedEnd.bam"

static

final String umms.core.writers.PairedEndWriter.readCigarFlag ="rc"

static

final String umms.core.writers.PairedEndWriter.readStartFlag ="rs"

static

---

The documentation for this class was generated from the following file:

- java/umms/core/writers/PairedEndWriter.java


---

Generated on Fri Aug 15 2014 15:11:15 for ESAT by  

 1.8.7
